# Bilateral sactosalphinx and congenital adrenal hyperplasia: case report on two rare conditions in two virgin girls

**DOI:** 10.1186/s13052-021-01089-2

**Published:** 2021-06-29

**Authors:** Maria-Grazia Scarpa, Marianna Iaquinto, Daniela Codrich, Jürgen Schleef

**Affiliations:** Pediatric Surgery Department - Institute for Maternal and Child Health, IRCCS Burlo Garofolo, via dell’Istria, 65/1, Trieste, Italy

**Keywords:** Sactosalpinx, Hydrosalpinx, Pyosalpinx, Congenital adrenal Hyperplasia, Case report

## Abstract

**Background:**

Sactosalpinx means a collection of fluid (serum, blood or pus) in the fallopian tube. CAH (Congenital Adrenal Hyperplasia) is a typical 46XX DSD (Disorder of Sex Development) due to a steroidogenic enzymatic defect. Both conditions are rare and can lead to reduced fertility rate.

**Case presentation:**

We describe two post-menarche virgin girls with CAH who were hospitalized for acute abdomen due to laparoscopically confirmed sactosalpinx. Case 1 recovered after conservative management, case 2 after a second-look and bilateral salpingectomy. The first case consisted of right sactosalpinx and previous peritonitis reported; the second one of bilateral symptomatic pyosalpinx and previous vaginal stenosis. Recurrent abdominal pain persisted at follow-up in Case 1: post-operative MRI (Magnetic Resonance Imaging) showed bilateral hydrosapinx that disappeared at a following ultrasound scan control. Follow-up was uneventful 36 months after surgery in Case 2, except for the surgical revision of the vaginal introitus.

**Conclusions:**

CAH-sactosalpinx association is a very rare but not negligible event. We suggest a conservative approach for sactosalpinx if tubal and/or ovary torsion can be excluded. Pyosalpinx is more challenging to treat, but during pediatric age we suggest starting with a conservative approach, especially in patients with CAH who have a potential low fertility rate. Careful gynecological follow-up after menarche is recommended to rule out any further causes of infertility.

## Background

Congenital Adrenal Hyperplasia (CAH) and sactosalpinx, are two rare conditions that adversely affect future fertility in girls.

CAH is a typical 46XX Disorder of Sex Development (DSD) due to a steroidogenic enzymatic defect. The most common form is the classic CAH due to 21-hydroxylase deficiency: an excessive adrenal androgen biosynthesis results in masculinization of the genitalia in XX offspring [1] and lead to ambiguity of the external genitalia and salt wasting syndrome. Another less severe form is characterized by virilization without salt waste.

17-OH-hydroxyprogesterone serum increase and genital inspection lead to diagnosis.

Prenatal androgen stimulation is responsible for the presence of UGS (Urogenital Sinus) and clitoris hypertrophy at birth in the most of female fetus. In 46 XX newborn, genital ambiguity at birth varies from near female-like anatomy to near male-like (stage 1 to 5 according to Prader score) [2].

Medical treatment involves the administration of steroid drugs associated with mineralocorticoids or not.

Surgical treatment involves female genitoplasty (FG) consisting of UGS correction by total or partial mobilization [3–5] or by particular surgical techniques like ASTRA (Anterior Saggital Transrectal Approach) [6] or laparoscopic assisted vaginal pull-through [7], so that complex flaps, urethro-vaginal complete separation, and vaginal pull-throughs under tension are mostly avoided in modern FG [8]. FG even includes clitoroplasty and reconfiguration of labia minora and majora. Timing of FG is still controversial. Vaginal stenosis is a possible complication [8].

Both pelvic surgery and vaginal stenosis and are risk factors for sactosalpinx.

We describe two cases of sexually inactive post-menarche girls with CAH and symptomatic sactosalpinx.

Both cases needed diagnostic laparoscopy for diagnosis confirmation.

We discuss the “*watch and wait*” approach for uncomplicated cases: patients and parents/guardians must be adequately informed about the disease, *pros* and *cons* and potential risks of conservative or surgical treatment.

Girls affected by CAH and sactosalpinx need a rigorous clinical and ultrasound follow-up to achieve the best chances of fertility and the lowest risk of complications.

## Cases presentation

### Case 1

A 14-years-old virgin girl was admitted to our Emergency Room for acute abdomen symptoms (one-day history of lower quadrant acute pain not responsive to analgesic, associated with fever and vomiting). Patient history was significant for diagnosis of CAH when she was 2-months-old, laparotomy and appendicectomy for complicated appendicitis when she was 8 years old, FG 1 year later. Both interventions were performed at another Hospital in Romania (no documentation available regarding the operations). Perineal examination before the onset of sactosalpinx showed: clitoromegaly, regular urethral meatus, no vaginal stenosis at vaginoscopy; no urinary incontinence, nor urinary tract infections. Menarche at the age of 12, regular menses, history of recurrent lower abdominal and ultrasonographic findings of regular ovaries and uterus. She irregularly took oral therapy with hydrocortisone (at dose of 19,7 mg/mq) and Florinef®: 0,1 mg/day.

**Fig. 1:**
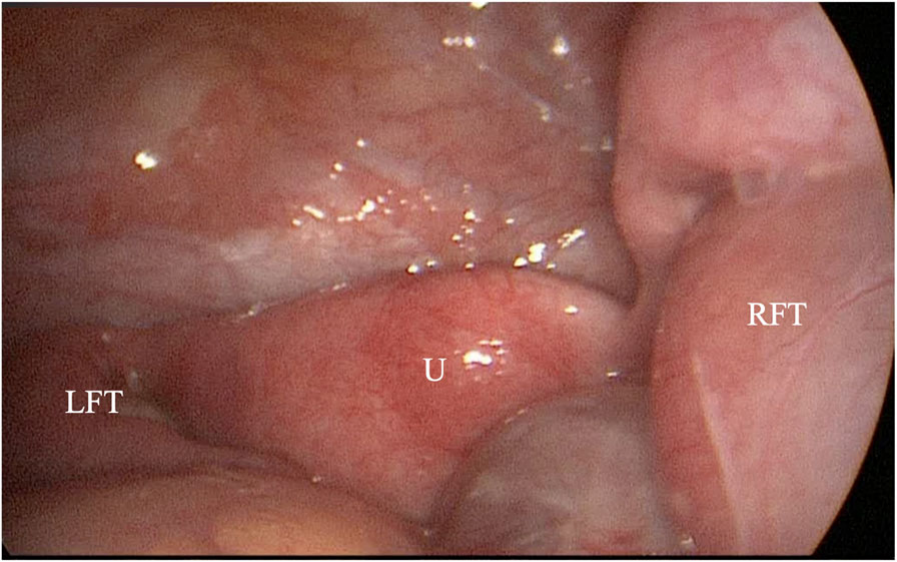
Case 1: laparoscopic view of bilateral hydrosalpinx. U = uterus; RFT = right fallopian tube (gross dilatation); LFT = left fallopian tube (moderate dilatation)

At admission, clinical examination revealed tenderness with guarding in pelvic region especially in the right iliac fossa. Serology revealed white cell count of 18.640 μL with neutrophilia and CRP (C-Reactive Protein) of 2,8 mg/dL at admission increased up to 16.4 mg/dL. An US (Ultrasound Scan) showed a large multilocular tubular fluid collection consistent of tube-ovarian structure (diameter more than 9 × 4.7 cm) and presence of liquid film in pelvic cavity. Urgent diagnostic laparoscopy was performed, because acute abdominal pain persisted and ovary and/or tubal torsion could not be excluded. Intra-operatively, right hydrosalpinx with a gross tubular dilatation was found without adnexal torsion, (see Fig. 1): fallopian tube dilatation was secondary to an extrinsic cause (peritoneal adhesions after previous laparotomy). Post-operative abdominal adhesions and a fluid collection inside pelvic cavity were also found (no bacteria at culture examination). Considering patient’s age and the underlying condition (CAH) it was decided for conservative management, after consultation with gynecologists. Parenteral therapy with ceftriaxone, metronidazole and doxiciclin started and continued for 10 days. Postoperative course was uneventful.

**Fig. 2:**
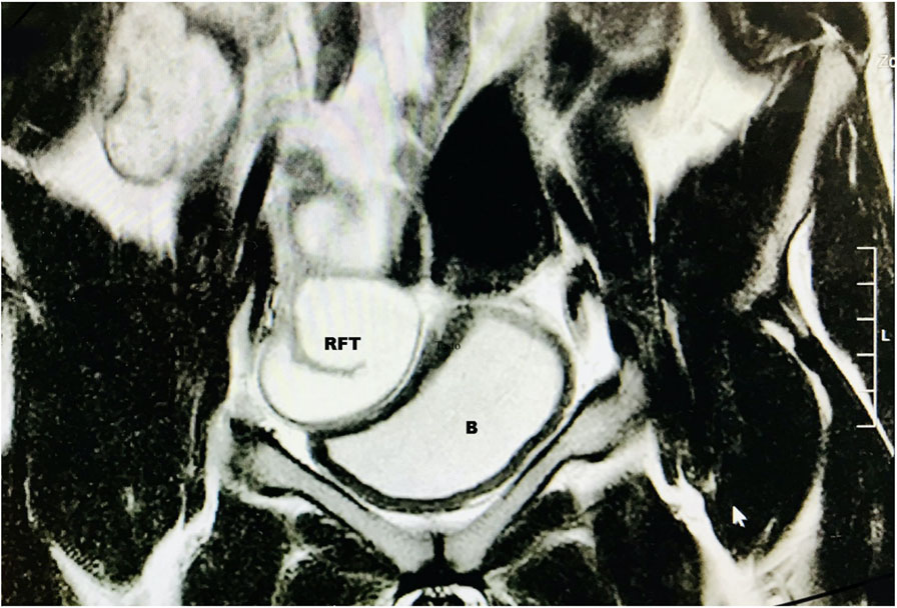
Coronal scan of pelvic MRI showing hydrosalpinx in case 1. B = bladder; RFT = right fallopian tube (C-shaped dilated tubular structure)

Recurrent abdominal pain persisted at follow-up: 9 months later, the post-operative MRI (Magnetic Resonance Imaging) showed bilateral hydrosapinx (see Fig. 2) that disappeared at a following ultrasound scan control 10 months later.

### Case 2

A not sexually active 15-years-old girl was evaluated for acute abdomen and fever.

Patient history was significant for diagnosis of CAH at birth, FG performed at our Institute when she was 21-months-old (high common channel used for urethra, flap vaginoplasty, clitoroplasty and labioplasty), surgical treatment for vaginal stenosis when she was 12-years-old and 16-years- old. Before the onset of acute symptomatology, the patient had recurrent low urinary tract infections history, no urinary incontinence. Urethral meatus was near clitoris (no clitoromegaly), vaginal introitus was narrow: its surgical revision and vaginal dilatation were performed 3 year before the acute symptoms occurred. She referred menarche at the age of 14 and regular menses. Ultrasound scan was significant for regular ovaries and uterus deflected to left. The patient regularly took oral therapy with hydrocortisone (at dose of 18,09 mg/mq) and Florinef®: 0,2 mg/day.

Physical examination, ultrasound scan and blood examination at admission suggested bilateral pyosalpinx. An urgent diagnostic laparoscopy was performed at another Hospital and confirmed the diagnosis. It was decided for conservative management even in this second case. The girl started standard parenteral antibiotic therapy for pyosalpinx but symptoms persisted and bilateral salpingectomy was necessary at the second-look laparoscopy 2 weeks later. Post-operative course was regular. A second surgical operation to enlarge vaginal introitus was needed after 1 year. Follow-up was then uneventful after 3 years.

## Discussion and conclusion

Sactosalpinx is a rare cause of abdominal pain in pediatric age and the clinical picture range from asymptomatic form to acute abdomen.

It occurs when a distally blocked fallopian tube fills with fluid collection of serum (hydrosalpinx), blood (hematosalpinx) or pus (pyosalpinx).

Occlusion of the fimbriated end of the tube may lead to tubal dilatation, usually in the ampulla and infundibulum. As far as the etiology is concerned, primary causes involve the anatomy, like abnormalities of length, mobility and structure. Secondary causes are acquired. The most common cause of hydrosalpinx is pelvic inflammatory disease, other causes include endometriosis, peri-tubal adhesion from a previous operation, tubal cancer and tubal pregnancy [9]. These secondary causes are typical of the adult age. Hydrosalpinx is far less common in children and adolescent. In pediatric age the pathogenesis remain ill-defined [10]. It has been strongly suggested that in younger non-sexually active women, a hydrosalpinx occurs secondary to a developmental defect [11]. A systematic review in 2015 found 66 total cases described in literature and demonstrated comparable outcomes between surgical, medical and conservative management [12]. In this review an association was found with: congenital hydrocolpos/hydrometra, imperforate anus; congenital fallopian atresia; imperforate hymen; Herlyn-Werner-Wunderlich syndrome; Mayer-Rokitansky-Küster-Hauser syndrome; Hirshsprung’s disease; persistent urogenital sinus; bicornate uterus/septate vagina; previous abdomino-pelvic surgery; complex genitourinary malformation (Müllarian abnormality).

US and MRI guide for diagnosis. On MRI, hydrosalpinx appears as a fluid-filled C- or S-shaped tubular structure that arise from the upper lateral margin of the uterus [9], but often definite diagnosis requires an explorative laparoscopy or laparotomy.

CAH is the most common cause of 46XX-DSD due to fetal androgen exposure [13]. It is an autosomal recessive disorder involving synthesis of cortisol. The most common form is the classic form due to 21-hydroxylase deficiency: an excessive adrenal androgen biosynthesis results in masculinization of the genitalia in XX offspring [1] and lead to ambiguity of the external genitalia and salt wasting syndrome. Just virilizing forms without salt waste exist. Concerning female newborn, karyotype (46XX), increase of 17-OH-hydroxyprogesterone and genital inspection may lead the diagnosis. Replacement therapy with steroid and often mineralocorticoid drugs is required. To conform genital anatomy to the sex of rearing, FG is often required (timing is still controversial). Clitoris and UGS surgery consist of an appropriate reconstructive procedure that should be individualized according to patient genital anatomy. FG generally includes urethroplasty, vaginoplasty, clitoroplasty and labioplasty.

The most challenging part of this intervention is the separation of the vagina from the urethra, especially in cases with a high confluence (UGS length more than 3 cm). Besides the technical difficulty, the patient is at risk for post-operative urinary incontinence, urethra-vaginal fistula and vaginal or urethral stenosis [3].

To facilitate reconstruction and decrease the risk of complications, partial UGS mobilization was described [4, 5]. Rink et al. [4] applied this technique to children with CAH through a perineal prone approach. It also allowed *en bloc* mobilization of the urethrovaginal confluence, with repositioning of the urethral and vaginal orifices in a separate orthotopic location.

The goal of surgical reconstruction for girls with CAH and UGS are recreating normal appearing and functional external genitalia, preserving bladder function, and maximizing the potential for a normal sexual and reproductive adult life. This goal may be technically harder to achieve in children with a high urethra-vaginal confluence and higher degrees of virilization.

CAH and sactosalpinx are two rare in pediatric age and they both lead to reduced fertility rate.

In 2011 Boukaidi et al. [14] reported 6 cases of isolated tubal torsion associated with hydrosalpinx. Salpingectomy were required in 5/6 patients. The histopathologic assessment of the resected tubes showed remaining ciliated cells in 50% of cases suggesting the possibility for tubal conservation.

Both our cases had predisposing factors to sactosalpinx: Case 1 had peritoneal adhesions occluding the fallopian right tube due to previous surgery (laparotomy for peritonitis); Case 2 had a history of vaginal stenosis and recurrent urinary infections .

We recommend a conservative approach for asymptomatic hydrosalpinx and for symptomatic sactosalphinx in general, except for septic cases or no responder patients (worsening symptoms despite adequate antibiotic treatment). If symptoms are controlled by analgesic/antibiotic therapy and adnexal torsion or other associated conditions (neoplasm) are excluded at surgery, we encourage a conservative treatment and a strict clinical and radiological follow-up: ultrasound scan every 6–12 months until adult age and periodical genital visit under anesthesia to exclude predisposing factor like vaginal stenosis for CAH surgically treated patients.

In our opinion, especially in cases with reduced fertility potential, it is important not to neglect predisposing conditions like:

1. Vaginal stenosis, particularly when associated with recurrent urinary infections
2. Previous pelvic surgery or previous laparotomy/laparoscopy for peritonitis for example due to complicated appendicitis or other abdominal diseases.

## Data Availability

The dataset supporting the conclusions of this article is included within the article.

## Abbreviations

CAH: Congenital Adrenal Hyperplasia
DSD: Disorder of Sex Development
MRI: Magnetic Resonance Imaging
UGS: Urogenital sinus
FG: Female Genitoplasty
ASTRA: Anterior Saggital Transrectal Approach
CRP: C-Reactive Protein
US: Ultrasound Scan
